# *Bacillus subtilis* Biofilm Development – A Computerized Study of Morphology and Kinetics

**DOI:** 10.3389/fmicb.2017.02072

**Published:** 2017-11-07

**Authors:** Sarah Gingichashvili, Danielle Duanis-Assaf, Moshe Shemesh, John D. B. Featherstone, Osnat Feuerstein, Doron Steinberg

**Affiliations:** ^1^Biofilm Research Laboratory, Institute of Dental Sciences, Faculty of Dental Medicine, Hebrew University-Hadassah, Jerusalem, Israel; ^2^Department of Prosthodontics, Faculty of Dental Medicine, Hebrew University-Hadassah, Jerusalem, Israel; ^3^Department of Food Quality and Safety, Institute for Postharvest Technology and Food Sciences, Agricultural Research Organization (ARO), The Volcani Center, Bet Dagan, Israel; ^4^School of Dentistry, University of California, San Francisco, San Francisco, CA, United States

**Keywords:** *Bacillus subtilis*, biofilms, growth kinetics, colony morphology, image processing, computer-assisted

## Abstract

Biofilm is commonly defined as accumulation of microbes, embedded in a self-secreted extra-cellular matrix, on solid surfaces or liquid interfaces. In this study, we analyze several aspects of *Bacillus subtilis* biofilm formation using tools from the field of image processing. Specifically, we characterize the growth kinetics and morphological features of *B. subtilis* colony type biofilm formation and compare these in colonies grown on two different types of solid media. Additionally, we propose a model for assessing *B. subtilis* biofilm complexity across different growth conditions. GFP-labeled *B. subtilis* cells were cultured on agar surfaces over a 4-day period during which microscopic images of developing colonies were taken at equal time intervals. The images were used to perform a computerized analysis of few aspects of biofilm development, based on features that characterize the different phenotypes of *B. subtilis* colonies. Specifically, the analysis focused on the segmented structure of the colonies, consisting of two different regions of sub-populations that comprise the biofilm – a central “core” region and an “expanding” region surrounding it. Our results demonstrate that complex biofilm of *B. subtillis* grown on biofilm-promoting medium [standard lysogeny broth (LB) supplemented with manganese and glycerol] is characterized by rapidly developing three-dimensional complex structure observed at its core compared to biofilm grown on standard LB. As the biofilm develops, the core size remains largely unchanged during development and colony expansion is mostly attributed to the expansion in area of outer cell sub-populations. Moreover, when comparing the bacterial growth on biofilm-promoting agar to that of colonies grown on LB, we found a significant decrease in the GFP production of colonies that formed a more complex biofilm. This suggests that complex biofilm formation has a diminishing effect on cell populations at the biofilm core, likely due to a combination of reduced metabolic rate and increased levels of cell death within this region.

## Introduction

Biofilms are surface-bound bacterial communities where bacteria co-exist embedded in an extra-cellular matrix (Flemming and Wingender, 2010). It has been shown that this type of organized structure facilitates bacterial survival in extreme pH, nutrient-poor, or otherwise hostile environments (Donlan and Costerton, 2002). Biofilm formation was also shown to aid bacteria in long-term adhesion to liquid interfaces or solid surfaces, which further adds to bacterial resistance against antibacterial agents (Donlan, 2002).

The Gram-positive, rod-shaped bacterium *Bacillus subtilis* is usually found in soil and is believed to be a commensal species of the human gastrointestinal tract (Hong et al., 2009). *B. subtilis* is considered to be non-pathogenic to humans and was shown to be beneficial to plants when in association with plant roots (Chen et al., 2013). The species is widely used in microbiology research and is considered to be a facile model organism for the study of biofilms, particularly due to its ability to form distinctly segmented three-dimensional colony biofilms (Bridier et al., 2013). Under conditions of stress, *B. subtilis* forms endospores that can withstand extreme environmental conditions for prolonged periods of time, thus enabling the survival of the organism under conditions such as nutrient depletion or under other various unfavorable environments (Nicholson et al., 2000).

It has been shown that lysogeny broth (LB) growth medium enriched with glycerol and manganese (LBGM) promotes *B. subtilis* biofilm formation (Shemesh and Chai, 2013). At the same time, high concentrations of Mg^2+^ ions in the medium were shown to have an inhibitory effect on biofilm growth (Oknin et al., 2015). Three main cell phenotypes were identified in *B. subtilis* colony type biofilm formation: motile, matrix-producing, and spore-forming (Vlamakis et al., 2013). Such phenotypic differentiation may contribute to *B. subtilis*’ ability to form uniquely segmented biofilms that consist of visibly different regions of sub-populations of cells. In particular, colony type biofilm grown under biofilm promoting conditions such as LBGM has a visually distinctive appearance from its standard LB counterpart (Shemesh and Chai, 2013). In LBGM, the colony biofilm is thicker and includes a central region which is characterized by a complex network of channels rich in matrix-producing bacterial cells, a region that is also associated with active sporulation and cell death (Vlamakis et al., 2013; Oknin et al., 2015). Morphology of the central region of the macrocolony biofilm is characterized by the presence of crisscrossing channels of live bacteria which can be seen as “wrinkles” in the biofilm (Bridier et al., 2011). Visible on the surface of the biofilm in whole colony imaging, these wrinkles can be identified in two-dimensional images as a network of distinctive bands at the center of the colony (Duanis-Assaf et al., 2015). On a molecular level, Shemesh and Chai (2013) showed that such biofilm is also characterized by an increased matrix production and sporulation. Actively sporulating and rich in matrix-producing cells, *B. subtilis* biofilms are often characterized as “mature” biofilms in the literature and referred to as being more complex and developed – features which are indicative of robust biofilms that are less susceptible to detrimental treatments.

Bacterial biofilm colonies can be characterized by their composition (cells and extra-cellular substances) and structure (proportions, spatial distribution, surface adherence). A characterization of “biofilm complexity” or “robustness” can be derived from its basic physical features such as thickness, size, and shape. Additionally, spatial distribution characteristics (e.g., uniform vs. segmented morphology) of the colony biofilm, dependent on environmental conditions, may also be indicative of key bacterial community properties such as strain pathogenicity (Costerton et al., 1999) and susceptibility to treatments (Stewart, 2003).

Several computerized techniques for structural biofilm analysis have been proposed based on various types of imaging methods. For example, Xavier et al. (2003) developed an automated biofilm morphology software toolbox based on three-dimensional confocal laser scanning microscopy (CLSM) images, which allows the automated quantification of such features as area of microbial colonization, biovolume, colony height, and more. Renslow et al. (2011) used computerized biofilm binary image reconstructions to compare such structural parameters as cell cluster shapes and their spatial relations within the biofilm. Bridier et al. (2010) performed a three-dimensional computerized analysis of 60 opportunistic pathogens with biovolume, thickness, substratum coverage, and roughness values for each.

However, the abovementioned computational approaches, while being useful tools for assessing general biofilm features that are common to multiple bacterial species, lack the ability to model features that are specific to a particular colony type biofilm such as that of *B. subtilis*. In particular, the non-uniform structure of *B. subtilis* biofilm, caused in part by varying regional patterns of cellular differentiation (Vlamakis et al., 2008), requires a custom computational model that takes into account those variations in colony structure. Furthermore, few computational models are available of whole colony growth as a function of time and existing approaches to morphology analysis of bacterial colonies tend to focus on small cross-section samples of colony type biofilms.

We hereby present a comparative analysis, specifically designed for *B. subtilis* matrix-producing phenotypes that form colony type biofilms, based on the analysis of fluorescently marked growing colony biofilms. Our model takes into account regional differences that are visible in *B. subtilis* colony type biofilms and enables whole colony characterization under different growth media consistencies over time. While the methodology presented in this paper was developed specifically for *B. subtilis* colony type biofilms, we would like to note that it can be extended to other organisms that form complex non-uniform structures. Most similar in overall macrocolony structure to *B. subtilis* are certain biofilm phenotypes of *Staphylococcus aureus* (Koch et al., 2014), *Escherichia coli* (Gomez-Gomez and Amils, 2014) and *Vibrio fischeri* (Ding et al., 2016) in which very similar visually distinct regions are apparent.

## Materials and Methods

### Strain and Growth Media

Starter cultures of *B. subtilis* YC161 (*P*_spank_*-gfp*) (Chai et al., 2011) were grown in LB [10 g of tryptone (Neogen, Lansing, MI, United States), 5 g of yeast extract (Neogen, Lansing, MI, United States), and 5 g of NaCl per liter] and incubated at 37°C at 150 *rpm* for 5 h. The LB medium was solidified by addition of 1.5% (w/v) agar, a standard methods agar value recommended by the American Public Health Association (Neogen, Lansing, MI, United States). LBGM media was prepared as described previously by supplementing LB with 1% (v/v) glycerol and 0.1 mM MnSO_4_ (Shemesh and Chai, 2013).

### Biofilm Formation

For colony type biofilm formation starter cultures were prepared as described above. The 2.5 μl of suspension from the starter culture [OD (600 nm) = 1] was placed onto agar plates prepared from different media. The plates were incubated at 30°C for a week. Images of the different sized colony type biofilms were taken every 24 h using Nikon SMZ25 microscope with ORCA-R2 camera (Zeiss LSM510 CLS microscope, Carl Zeiss, Oberkochen, Germany). All images were taken using objective magnification of 1 and an exposure time of 100 ms.

### Multi-Stain Confocal Visualization of Bacterial Viability

Bacterial biofilms were removed from agar surfaces to glass cover slips using phosphate-buffered saline (PBS). The colonies were incubated with 700 μl mix dye of propidium iodide (PI) stain for labeling dead bacteria and Concanavalin A (Con A) Alexa Fluor 647 for labeling extra-cellular polysaccharides (EPS) for 20 min at RT and afterward washed using PBS. All images were taken using Zeiss LSM510 CLS microscope (Carl Zeiss, Oberkochen, Germany). PI fluorescence was measured using 543 nm excitation and 570 nm emission. Alexa Fluor 647 was measured using 650 nm excitation wavelength and 668 nm emission (Assaf et al., 2015; Feldman et al., 2016). Three-dimensional images of colony type biofilms’ core regions were constructed using Zen software (Carl Zeiss). At least three random fields were observed and analyzed in five independent experiments. For negative controls, *B. subtilis* non-labeled strain NCIB3610 was used to exclude possibility of fluorescence being emitted from sources other than the bacterial cells. Additionally, *B. subtilis Δeps* (RL3852) strain was used for negative control of EPS staining by Con A (Kearns et al., 2005).

### Image Processing

All images were saved in Portable Network Graphics (PNG) format and analyzed in MATLAB R2015a (The MathWorks, Inc., 2015). Images were of equal size and were not normalized in any way for our calculations. For one-dimensional intensity signal analysis, the images were converted to grayscale by taking the mean value of three color channels in RGB color space. In such grayscale representation, strong GFP signal is seen as white pixels in the image, whereas areas with non-viable bacteria or areas with no bacterial presence appear as black pixels.

#### Biofilm Growth Kinetics Analysis

**FIGURE 1:**
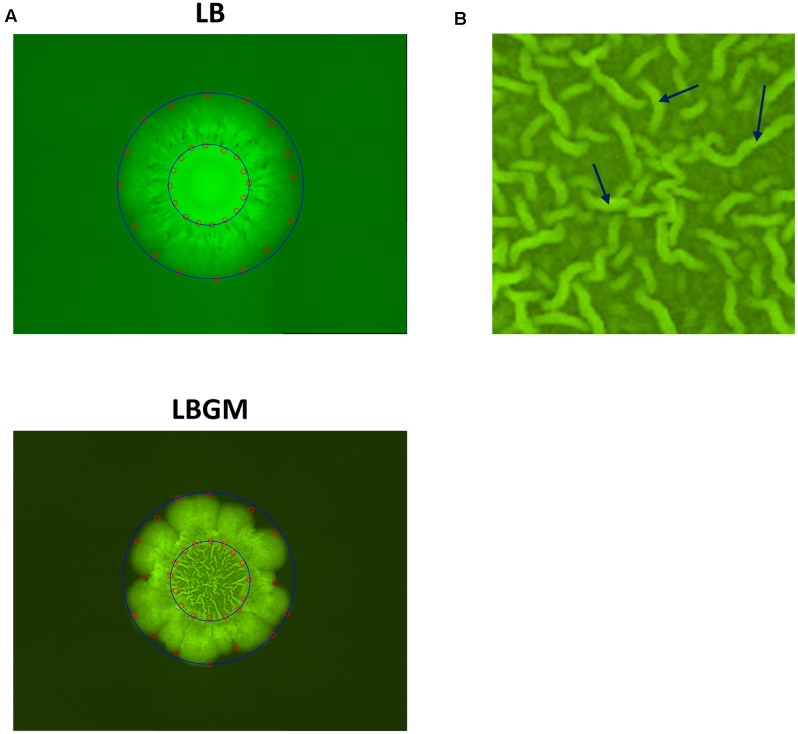
Colony segmentation. **(A)** LB (top) and LBGM (bottom) colony type biofilm images with marked points along the inner and outer boundaries (red). Minimal bounding circles of the “core” and “expanding” regions are shown (blue). Images were taken after 72 h of growth. **(B)** Close-up of biofilm core grown on LBGM. A network of channels can be seen, with several individual bands marked with arrows.

In order to assess the growth kinetics of bacterial colonies, we measured two types of distances in each image. The average radius of the whole colony was measured as the average Euclidean distance of 16 manually selected points on the outer boundary of the colony from the center of the image (**Figure 1A**). By sampling four distinct points in each quarter of an image (and thus 16 overall), it is possible to account for the asymmetry and variations that occur during biofilm development along the region boundaries. Similarly, 16 points along the inner colony core boundary were used to measure the inner core radius. Inner core area in biofilm images was further defined as the area of the minimal bounding circle that contains all 16 points along the inner boundary (**Figure 1A**). The average grayscale intensity value of all pixels within this area is then used as a measurement of mean GFP signal of the colony core. Likewise, whole colony area can be defined by a larger bounding circle which contains all points along the outer boundary.

#### Intensity-Based Measure of Biofilm Complexity

Our characterization of biofilm robustness or complexity is based on a measurement of signal convolutedness, which relies on the three-dimensional complex nature of intensities at the biofilm core. We quantify this complex behavior by analyzing intensity values along a spiral path through the colony core, which traverses the image starting from its center toward the periphery. The length of the spiral path was set to 2000 pixels, a value that sufficiently covers a central region of the colony core in the image (**Figure 6A**). Intensity values along the abovementioned spiral path were then plotted as a one-dimensional signal and analyzed as a two-variable function:

• Changes in signal amplitude can be used to determine the magnitude of crossovers between newly formed bands of live bacteria and deeper layers of the biofilm, which consist of aged bacterial cells that no longer produce a comparable GFP signal to that produced by newly formed cells. The amplitude is defined as the absolute difference between the maximum and minimum values of the signal. However, in order to take into account the fluctuations that occur along the entire signal, we calculated the signal amplitude as the average amplitude of consecutive overlapping local windows of 20 pixels along the signal.
• Changes in signal frequency components can be used to determine the three-dimensional complex nature of the GFP signal obtained from the colony biofilm core. As the biofilm matures, bacterial bands that characterize the biofilm core become thinner and more dense within the central region of the colony biofilm. Consequently, the changes in amplitude become more and more frequent as crossovers between newly formed bands of bacteria and deeper biofilm layers occur with increasing frequency along a cross spiral cut through the biofilm core. The frequency value of a signal was calculated (similarly to the signal amplitude) as the average statistical variance of intensity values in consecutive overlapping local windows of 20 pixels along the signal.

### Statistical Analysis

The data obtained were analyzed statistically using ANOVA following *post hoc T*-test with Bonferroni correction. All statistical analyses were performed using Microsoft Excel software. Data represent 11 biological repeats for samples grown on LB agars and 8 biological repeats for those grown on LBGM agars.

## Results

**FIGURE 2:**
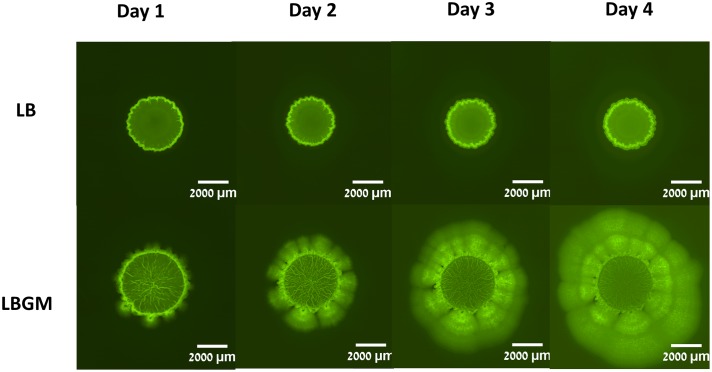
Colony biofilm formation by *Bacillus subtilis. B. subtilis* cultures harboring *P*_spank_*-gfp* (YC161) were seeded on LB agar plates or LB agar plates supplemented with glycerol and manganese (LBGM). The biofilms were grown at 30°C for 4 days. Images of the entire colony biofilm were taken using Nikon SMZ microscope with GFP filter, every 24 h.

Colony type biofilms of *B. subtilis*, grown on two different LB-based agar media, were shown to exhibit different growth and organization profiles. Significant differences were observed in the growth kinetics, bacterial long-term GFP production, and morphological features of the colony type biofilms. As can be seen in **Figure 1A**, both LB- and LBGM-type colonies are characterized by a central core, surrounded by a prominent peripheral area – both core and periphery are shown circumscribed in **Figure 1A** by circular boundaries (blue). The most noticeable difference between the colonies is the complexity of the inner core – while the core of a biofilm grown on LB medium is smooth and uniform in pixel intensity, its LBGM counterpart appears to contain a network of high-intensity ribbons that crisscross one another throughout the center of the biofilm (**Figure 1B**). These structures, which can be seen forming in biofilms over time (**Figure 2**), were determined to be an interconnected network of hollow channels, which enhance nutrient transport across the biofilm (Wilking et al., 2013). We now proceed to describe the results of our computerized analysis which are based in part on the definitions of biofilm “core” and “periphery,” as defined above.

### Colony Growth Kinetics

**FIGURE 3:**
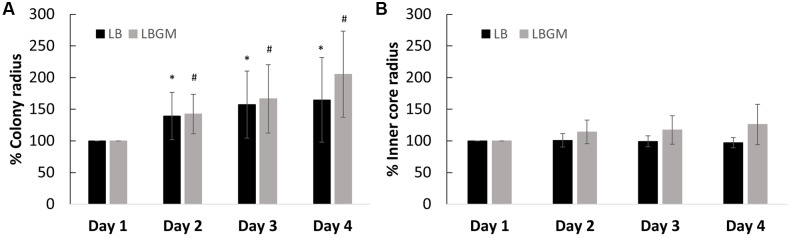
Growth kinetics of *B. subtilis* biofilm colonies. Radii of the growing biofilm colonies were measured over a period of 4 days. The graphs above represent change in radii for both groups (LB and LBGM), respective to the starting radius of each group on day 1 (100%) – of the entire colony **(A)** and of the colony core **(B)** on LB and LBGM agars. The values represent the mean and SD values of 11 samples for LB and 8 samples for LBGM agars. Analysis was done using ANOVA following *post hoc T*-test with Bonferroni correction. ^∗^*P*-value < 0.05, ^#^*P*-value < 0.01.

Colonies grown on LB and LBGM agars were characterized by varying expansion behaviors. Firstly, *B. subtilis* biofilms grown on LBGM showed stronger overall expansion ability over a period of 4 days, measuring on day 4 on average over twice the radii of the original colony radius on day 1 (205%) compared to bacterial colonies grown on non-biofilm promoting media (164%) (**Figure 3A**).

Secondly, the developing colonies were observed to be segmented into two main sections; a central core, in which a network of interconnected channels are formed under biofilm-promoting conditions and colony periphery, which appears to enclose the colony and separate it from its environment (**Figure 2**). A visible circular band can often be seen partitioning the two regions and while the differences between the two are most striking in colonies grown on LBGM, the same phenomenon can be seen in biofilms grown on LB as well (**Figure 1A**). We were able to separately analyze the growth kinetics of the entire colony and of the central “core” region using a computerized approach, as described in the section “Materials and Methods” (section “Biofilm Growth Kinetics Analysis”). Briefly, by manually placing a series of markers around the regions of interest, we were able to measure the average Euclidean distances from the center of the colony to the pre-set markers (**Figure 1A**). Since the colonies are symmetrical in nature, we calculated the average of those distances to approximate the respective radii of the inner core and the whole colony.

Our results show that the overall increase in colony biofilm size is mostly attributed to the growth of the expanding outer section rather than the inner core, which in contrast, does not appear to change significantly in size. In colonies grown on LB, over the course of 72 h, the inner core radius does not diverge by more than 4% of its initial size (**Figure 3B**), while the entire colony expands to over 164% in radius (**Figure 3A**). A similar effect can be seen in colonies grown on LBGM, where the inner core size reaches a little below 126% in radius (**Figure 3B**), as opposed to the increase in overall colony radius (205%) (**Figure 3A**).

### Bacterial Long-Term Viability

**FIGURE 4:**
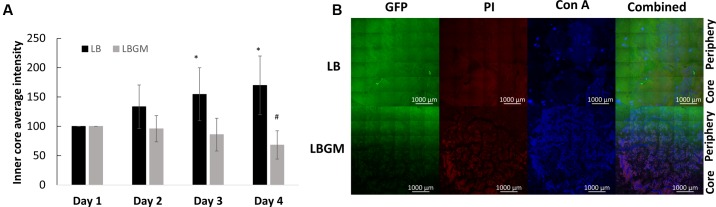
Long-term viability analysis of *B. subtilis* colony biofilm. **(A)** Core intensity of the formed biofilms was calculated as the average intensity of all pixels within circles of equal radii from the center of the colony. The graph represents the average intensity values of the biofilm core evaluated over 4 days of growth for LB and LBGM agars, with statistical analysis done for both groups with respect to the starting intensity of each group on day 1 (100%). The values represent the mean and SD values of 11 samples for LB and 8 samples for LBGM agars. Analysis was done using ANOVA following *post hoc T*-test with Bonferroni correction. ^∗^*P*-value < 0.05, ^#^*P*-value < 0.01. **(B)** A representative CLSM image of the boundary between colony core and periphery. In the combined LBGM image, the boundary can be clearly seen as a noticeable separation between a lower region that is characterized by weak GFP signal and is mostly dominated by PI and Con A staining (Alexa Fluor 647) and the area right above it that contains stronger GFP signal indicating living cells. Images taken after 4 days of colony growth.

**FIGURE 5:**
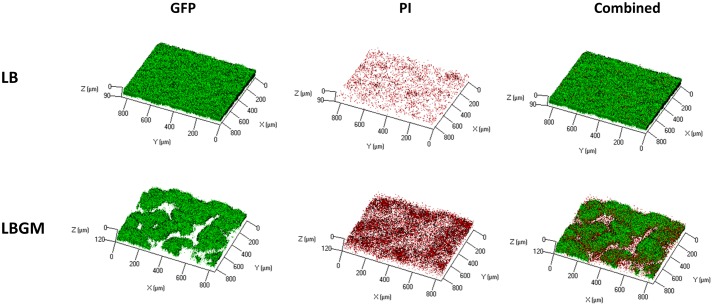
Live dead staining of complex *B. subtilis* colony biofilm core. CLSM imaging of inner core of *B. subtilis* biofilm following 4 days incubation on solid LB and LBGM media. GFP (left column) marks live bacterial cells, PI (center column) indicates the presence of dead cells. Right column shows a combination of the two colors. Images are representative of five independent experiments.

**Figure 4A** shows an analysis of bacterial GFP production over time, within the inner core region of the colony, measured as the average intensity value of the GFP signal within the colony core. A statistically significant decrease in intensity values was observed on day 4 in colonies grown on LBGM agar – over 30% decrease compared to same mean intensity measured on day 1. In contrast, colonies grown on LB only increased on average in mean inner core intensity values, by almost 70% (**Figure 4A**) on day 4 compared to same values on day 1. The strong signal decrease at the center of the colony can be confirmed under CLSM imaging on day 4 (**Figure 4B**). As can be seen, the center of the LBGM colony is abundant in PI-stained bacteria (red), while the periphery is dominated by live cells (green), surrounded by EPS (blue). This effect is greatly diminished in an LB colony grown over the same period where stronger GFP signal is observed at the core in correlation with less EPS production as indicated by Con A staining. The confocal image in **Figure 5** further illustrates the differences in composition of a complex biofilm core on day 4 by showing increased PI staining (red) on LBGM medium, compared to PI staining present in a comparable LB colony core. Additionally, it can be seen that while LB is characterized by a uniform GFP signal, LBGM colonies consist of wrinkled formations that can be visualized on the three-dimensional images in **Figure 5**.

### Biofilm Complexity

**FIGURE 6:**
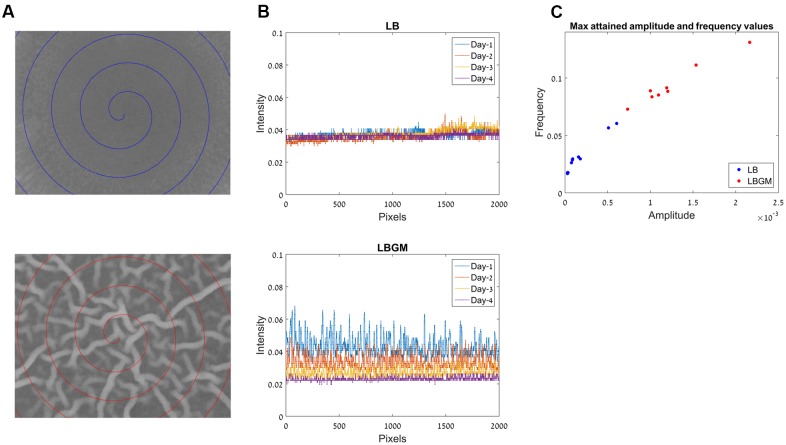
Frequency analysis for colony biofilm formation on LB and LBGM agars. **(A)** Center of the biofilm core in LB (top) and LBGM (bottom), after 72 h of growth. Spiral paths chart the pixel locations used in **(B)**. **(B)** A one-dimensional signal which comprises of pixel intensities along the spiral paths in **(A)**, in LB (top) and LBGM (bottom). **(C)** Maximal attained amplitude and frequency values for colonies grown on LB (blue) and LBGM (red).

Due to the varying phenotypes of *B. subtilis* colony type biofilms, it is often difficult to assess biofilm maturity in an objective manner. When visually comparing under-developed bacterial colonies (grown on LB) and complex biofilms (grown on LBGM), the differences are most striking within the colony cores (**Figure 2**). While the LB core remains smooth, corresponding to an under-developed and less-complex colony biofilm, LBGM core displays a clearly visible intercalated nature with bacterial bands interwoven in between the secreted matrix (**Figure 6A**).

Our computerized approach allows us to translate a complex image of the colony core into a one-dimensional signal that can be modeled by two variables – signal frequency and signal amplitude. Such dimensionality reduction is done by plotting intensities along a spiral path starting from the center of the image and moving outward with each 360° turn. This one-dimensional signal is shown in **Figure 6B**, for a colony grown on LB (top) and for a colony grown on LBGM (bottom). It can immediately be seen that over the entire growth period, the colony grown on LBGM is decreasing in both the amplitude and average intensity at the colony core (**Figure 6B** bottom). In comparison, a colony grown on LB exhibits a very slight increase in intensity on average, but is furthermore characterized by a much less pronounced amplitude and frequency over the same period (**Figure 6B** top).

**Figure 6C** shows the measured maximal amplitude and frequency values attained for colonies grown on LB (blue) and LBGM agars (red) over the entire growth period. It can be seen that the values in the LB and LBGM agars represent two separate clusters – LB colonies are located closer to the axis origin due to lower amplitude and frequency values, while LBGM colonies are characterized by greater values in both features. It can also be observed that there is significant variance of amplitude and frequency values within each cluster, corresponding to the variability of maximal complexity attained by the various colonies in our data set.

## Discussion

In this work, we describe a structure-preserving semi-quantitative computational method to assess biofilm formation using *B. subtilis* as a model organism. Specifically, we utilize a fluorescence microscope with camera attachment to monitor biofilm formation and development over time on two different solid agar surfaces – standard LB agar and LB agar supplemented with glycerol and manganese (LBGM). The supplemented LB agar is biofilm-promoting for *B. subtilis* colonies as reported by Shemesh and Chai (2013) who concluded that glycerol acts as a signal molecule for histidine kinase KinD, initiating a signal transduction pathway that leads to the formation of robust colony biofilm. Mn^2+^ molecules in the same pathway may be cofactors in the initial reaction or reactions further downstream.

Biofilm formation may undergo several morphological stages of maturity during growth. In *Bacillus* strains, the intercalated structures that are formed at the biofilm core usually after 2–3 days of growth are an indication of biofilm complexity and robustness (Branda et al., 2001). Under biofilm promoting conditions, such as a glycerol and manganese-rich growth medium, these structures are characterized by a high level of internal organization. In initial stages of development, wrinkled structures protrude from the periphery of the colony biofilm toward its core (Vlamakis et al., 2013). As the biofilm matures, these structures increase in number and density as the network of channels formed by bacterial cells at the center of the colony becomes more dense (**Figure 2**). Hence, complex *B. subtilis* biofilms in analyzed images are characterized by a highly complex structural organization of bacterial bands at the center of the colony.

*Bacillus* biofilms are characterized by non-random, distinct regions each governed by one of three different cellular phenotypes (Brehm-Stecher and Johnson, 2004; Veening et al., 2004). Under CLSM, as well as a fluorescent microscope, two such regions are clearly visible during development. The macro-colony core, which under LBGM is characterized by its internal mesh-like structure, and an outer region that surrounds the core and can be easily distinguished from it by a separating circular rim of high-intensity bacterial band. Presented analysis differentiates the inner core from an expanding region of the growing biofilm colony due to clearly visible differences in morphology that can be observed between the two areas in LB and LBGM agars (**Figure 1A**). Our computerized analysis focuses on three aspects of colony growth:

(a) rate measurement of colony growth/expansion
(b) measurement of long-term bacterial GFP production (both spatial and temporal differences)
(c) identification and modeling of key differences in morphology of complex and under-developed *B. subtilis* colony type biofilms.

Our results demonstrate that in both standard and biofilm-promoting conditions, biofilm growth occurs largely due to the expansion of its outer layers, while the size of the colony core, where the complex structural behavior is observed, remains predominantly unchanged (**Figure 3**). Localization of cellular phenotypes within the colony biofilm may explain the differences in expansion rates that were observed within colonies grown under biofilm-promoting conditions such as LBGM. Specifically, the outer rim of the macro-colony, which rapidly expands during development, is likely to contain a higher ratio of motile cells. This, in contrast to the macro-colony center, which is likely to be populated by a higher percentage of non-motile, sporulating, and dead cells (Asally et al., 2012).

Furthermore, we found that under biofilm promoting conditions the colony core is characterized by a significant decrease in long-term GFP production, as can be seen by the rapid weakening of emitted GFP signal when compared to that of colonies grown on LB medium for the same time period (**Figure 4A**). A significant decrease in signal intensity at the biofilm core was observed in LBGM colonies, while colonies grown on LB continued to hold or increase in their starting intensity values over the same time period. This result suggests that complex biofilm formation is correlated with rapid decrease in bacterial GFP production, suggesting that complex biofilm construction has an adverse effect on the metabolic activity and livelihood of bacterial cells located within the colony core. This result is further supported by the CLSM images (**Figure 4B**), which show a visible decrease in live GFP-producing bacterial cells at the center of the biofilm in LBGM colonies. In accordance with results by Shemesh and Chai (2013), who reported upregulated transcription of *epsA-O* operons in LBGM colonies compared to LB, we observed higher Con A staining signal in LBGM core which is associated with higher EPS production. **Figure 5** further demonstrates that LBGM biofilms are characterized by increased PI staining when compared to their LB counterparts. Since PI cannot freely penetrate cell membranes, its increased presence within the wrinkles suggests increased number of cells with structural membranal damage. It is worth noting that the decrease in GFP signal appears at the macro-colony center both in regions of highest structures at the biofilm core that can reach up to 300 μm in height (Bridier et al., 2011), as well as in deeper layers, as evidenced by a similar reduction in intensity that occurs in the background regions surrounding the high-intensity bands. Thus, the entire colony biofilm center is affected by biofilm formation. Such reduction in GFP signal can also be a direct effect of slowing of the cell metabolic rate at the center of the macro-colony, which in itself is a classical bacterial defense mechanism, and an indication of increased sporulation within that region. However, the uplift in PI staining leads us to conclude that there is involvement of cell death as well. This finding is consistent with Allocati et al. (2015), who report that cells in sporulating populations of *B. subtilis* undergo programmed cell death to release mature spores. Moreover, they find that sporulating cells are themselves involved in releasing killing factors, which cause non-sporulating cells in their vicinity to disintegrate.

Our model for assessing the complexity and robustness of the biofilm relies on the transformation of the developing colonies images into a one-dimensional signal, which is then evaluated via two scalar variables – signal amplitude and frequency. **Figure 6C** shows the distribution of those variable values for all colonies in our data set. Less complex colonies (grown on LB agar) can be seen occupying a region closer to the origin of the axes, while colonies that attained a more robust state during the same growth period (grown on LBGM) are characterized by higher amplitude and frequency values (red). By assigning each colony in the experiment amplitude and frequency values it is possible to not only clearly distinguish between clusters of colonies grown under different growth conditions and assess the magnitude of differences in their complexity, but also to compare and contrast colonies within the same cluster of growth conditions over a series of experiments.

Our proposed model is a non-disruptive approach for image-based analysis of *B. subtilis* colony type biofilms that enables us to characterize growth kinetics and changes in morphology that take place during colony development under different environmental conditions. The computerized analysis allows us to not only classify the various colony type biofilm phenotypes, but also to assess biofilm complexity and potentially predict bacterial pathogenicity. The process of colony formation can be numerically semi-quantified from microscopy images using objective structural parameters for biofilm image analysis, used to compare and monitor temporal variations in biofilm structure and metabolic activity over time. Eventually, the deciphering and classification of the colony macro-structure may allow us to derive additional properties of the biofilm such as distribution of mechanical forces (Asally et al., 2012), diffusion patterns (Lewis, 2001), and even areas of varying gene expression (Stewart and Franklin, 2008). Moreover, changes in biofilm morphology during various stages of maturity may affect its susceptibility to antibiotic treatments (Schultz et al., 2010).

## Author Contributions

SG together with DS planned the experiments and wrote the original manuscript. SG performed the experiments described in the manuscript. DD-A assisted in the experiments and setup. MS, OF, and JF revised the manuscript critically and provided academic guidance. SG and DS integrated all data in the study and penned the final manuscript.

## Conflict of Interest Statement

The authors declare that the research was conducted in the absence of any commercial or financial relationships that could be construed as a potential conflict of interest.

## References

[B1] Allocati N. Masulli M. Di Ilio C. De Laurenzi V. (2015). Die for the community: an overview of programmed cell death in bacteria. *Cell Death Dis.* 6 e1609. 10.1038/cddis.2014.570 25611384 PMC4669768

[B2] Asally M. Kittisopikul M. Rué P. Du Y. Hu Z. Çağatay T. (2012). Localized cell death focuses mechanical forces during 3D patterning in a biofilm. *Proc. Natl. Acad. Sci. U.S.A.* 109 18891–18896. 10.1073/pnas.1212429109 23012477 PMC3503208

[B3] Assaf D. Steinberg D. Shemesh M. (2015). Lactose triggers biofilm formation by *Streptococcus mutans*. *Int. Dairy J.* 42 51–57. 10.1016/j.idairyj.2014.10.008

[B4] Branda S. S. González-Pastor J. E. Ben-Yehuda S. Losick R. Kolter R. (2001). Fruiting body formation by *Bacillus subtilis*. *Proc. Natl. Acad. Sci. U.S.A.* 98 11621–11626. 10.1073/pnas.191384198 11572999 PMC58779

[B5] Brehm-Stecher B. F. Johnson E. A. (2004). Single-cell microbiology: tools, technologies, and applications. *Microbiol. Mol. Biol. Rev.* 68 538–559. 10.1128/MMBR.68.3.538-559.2004 15353569 PMC515252

[B6] Bridier A. Dubois-Brissonnet F. Boubetra A. Thomas V. Briandet R. (2010). The biofilm architecture of sixty opportunistic pathogens deciphered using a high throughput CLSM method. *J. Microbiol. Methods* 82 64–70. 10.1016/j.mimet.2010.04.006 20433880

[B7] Bridier A. Le Coq D. Dubois-Brissonnet F. Thomas V. Aymerich S. Briandet R. (2011). The spatial architecture of *Bacillus subtilis* biofilms deciphered using a surface-associated model and in situ imaging. *PLOS ONE* 6:e16177. 10.1371/journal.pone.0016177 21267464 PMC3022735

[B8] Bridier A. Meylheuc T. Briandet R. (2013). Realistic representation of *Bacillus subtilis* biofilms architecture using combined microscopy (CLSM, ESEM and FESEM). *Micron* 48 65–69. 10.1016/j.micron.2013.02.013 23517761

[B9] Chai Y. Norman T. Kolter R. Losick R. (2011). Evidence that metabolism and chromosome copy number control mutually exclusive cell fates in *Bacillus subtilis*. *EMBO J.* 30 1402–1413. 10.1038/emboj.2011.36 21326214 PMC3094124

[B10] Chen Y. Yan F. Chai Y. Liu H. Kolter R. Losick R. (2013). Biocontrol of tomato wilt disease by *Bacillus subtilis* isolates from natural environments depends on conserved genes mediating biofilm formation. *Environ. Microbiol.* 15 848–864. 10.1111/j.1462-2920.2012.02860.x 22934631 PMC3904073

[B11] Costerton J. W. Stewart P. S. Greenberg E. P. (1999). Bacterial biofilms: a common cause of persistent infections. *Science* 284 1318–1322. 10.1126/science.284.5418.1318 10334980

[B12] Ding Y. Tang J. Guo F. (2016). Identification of protein-protein interactions via a novel matrix-based sequence representation model with amino acid contact information. *Int. J. Mol. Sci.* 17:1623. 10.3390/ijms17101623 27669239 PMC5085656

[B13] Donlan R. M. (2002). Biofilms: microbial life on surfaces. *Emerg. Infect. Dis.* 8 881–890. 10.3201/eid0809.020063 12194761 PMC2732559

[B14] Donlan R. M. Costerton J. W. (2002). Biofilms: survival mechanisms of clinically relevant microorganisms. *Clin. Microbiol. Rev.* 15 167–193. 10.1128/CMR.15.2.167-193.2002 11932229 PMC118068

[B15] Duanis-Assaf D. Steinberg D. Chai Y. Shemesh M. (2015). The LuxS based quorum sensing governs lactose induced biofilm formation by *Bacillus subtilis*. *Front. Microbiol.* 6:1517. 10.3389/fmicb.2015.01517 26779171 PMC4705240

[B16] Feldman M. Ginsburg I. Al-Quntar A. Steinberg D. (2016). Thiazolidinedione-8 alters symbiotic relationship in *C. albicans*-*S. mutans* dual species biofilm. *Front. Microbiol.* 7:140. 10.3389/fmicb.2016.00140 26904013 PMC4748032

[B17] Flemming H.-C. Wingender J. (2010). The biofilm matrix. *Nat. Rev. Microbiol.* 8 623–633. 10.1038/nrmicro2415 20676145

[B18] Gomez-Gomez J. M. Amils R. (2014). Crowning: a novel *Escherichia coli* colonizing behaviour generating a self-organized corona. *BMC Res. Notes* 7:108. 10.1186/1756-0500-7-108 24568619 PMC3936827

[B19] Hong H. A. Khaneja R. Tam N. M. Cazzato A. Tan S. Urdaci M. (2009). *Bacillus subtilis* isolated from the human gastrointestinal tract. *Res. Microbiol.* 160 134–143. 10.1016/j.resmic.2008.11.002 19068230

[B20] Kearns D. B. Chu F. Branda S. S. Kolter R. Losick R. (2005). A master regulator for biofilm formation by *Bacillus subtilis*. *Mol. Microbiol.* 55 739–749. 10.1111/j.1365-2958.2004.04440.x 15661000

[B21] Koch G. Yepes A. Forstner K. U. Wermser C. Stengel S. T. Modamio J. (2014). Evolution of resistance to a last-resort antibiotic in *Staphylococcus aureus* via bacterial competition. *Cell* 158 1060–1071. 10.1016/j.cell.2014.06.046 25171407 PMC4163622

[B22] Lewis K. (2001). Riddle of biofilm resistance. *Antimicrob. Agents Chemother.* 45 999–1007. 10.1128/AAC.45.4.999-1007.2001 11257008 PMC90417

[B23] Nicholson W. L. Munakata N. Horneck G. Melosh H. J. Setlow P. (2000). Resistance of *Bacillus* endospores to extreme terrestrial and extraterrestrial environments. *Microbiol. Mol. Biol. Rev.* 64 548–572. 10.1128/MMBR.64.3.548-572.2000 10974126 PMC99004

[B24] Oknin H. Steinberg D. Shemesh M. (2015). Magnesium ions mitigate biofilm formation of *Bacillus* species via downregulation of matrix genes expression. *Front. Microbiol.* 6:907. 10.3389/fmicb.2015.00907 26441856 PMC4561805

[B25] Renslow R. Lewandowski Z. Beyenal H. (2011). Biofilm image reconstruction for assessing structural parameters. *Biotechnol. Bioeng.* 108 1383–1394. 10.1002/bit.23060 21280029 PMC3076525

[B26] Schultz G. Phillips P. Yang Q. Stewart P. (2010). Biofilm maturity studies indicate sharp debridement opens a time-dependent therapeutic window. *J. Wound Care* 19 320–328. 10.12968/jowc.2010.19.8.77709 20852503

[B27] Shemesh M. Chai Y. (2013). A combination of glycerol and manganese promotes biofilm formation in *Bacillus subtilis* via histidine kinase KinD signaling. *J. Bacteriol.* 195 2747–2754. 10.1128/JB.00028-13 23564171 PMC3697245

[B28] Stewart P. S. (2003). Diffusion in biofilms. *J. Bacteriol.* 185 1485–1491. 10.1128/JB.185.5.1485-1491.2003 12591863 PMC148055

[B29] Stewart P. S. Franklin M. J. (2008). Physiological heterogeneity in biofilms. *Nat. Rev. Microbiol.* 6 199–210. 10.1038/nrmicro1838 18264116

[B30] The MathWorks Inc. (2015). *MATLAB and Statistics Toolbox Release 2015a*. Natick, MA: The MathWorks, Inc.

[B31] Veening J.-W. Smits W. K. Hamoen L. W. Jongbloed J. D. Kuipers O. P. (2004). Visualization of differential gene expression by improved cyan fluorescent protein and yellow fluorescent protein production in *Bacillus subtilis*. *Appl. Environ. Microbiol.* 70 6809–6815. 10.1128/AEM.70.11.6809-6815.2004 15528548 PMC525234

[B32] Vlamakis H. Aguilar C. Losick R. Kolter R. (2008). Control of cell fate by the formation of an architecturally complex bacterial community. *Genes Dev.* 22 945–953. 10.1101/gad.1645008 18381896 PMC2279205

[B33] Vlamakis H. Chai Y. Beauregard P. Losick R. Kolter R. (2013). Sticking together: building a biofilm the *Bacillus subtilis* way. *Nat. Rev. Microbiol.* 11 157–168. 10.1038/nrmicro2960 23353768 PMC3936787

[B34] Wilking J. N. Zaburdaev V. De Volder M. Losick R. Brenner M. P. Weitz D. A. (2013). Liquid transport facilitated by channels in *Bacillus subtilis* biofilms. *Proc. Natl. Acad. Sci. U.S.A.* 110 848–852. 10.1073/pnas.1216376110 23271809 PMC3549102

[B35] Xavier J. White D. Almeida J. (2003). Automated biofilm morphology quantification from confocal laser scanning microscopy imaging. *Water Sci. Technol.* 47 31–37. 12701903

